# Quantitative evaluation of pigment epithelial encapsulation using polarization-sensitive optical coherence tomography after anti-VEGF therapy for myopic choroidal neovascularization

**DOI:** 10.1186/s40942-026-00902-6

**Published:** 2026-07-14

**Authors:** Mami Ota, Yuki Saeki, Kazuki Yashiro, Shuichiro Aoki, Kohdai Kitamoto, Ryo Terao, Keiko Azuma

**Affiliations:** 1 Department of Ophthalmology, The University of Tokyo, 7-3-1 Hongo, Bunkyo-ku, Tokyo, 113-8655 Japan; 2 Department of Ophthalmology, Graduate School of Medicine, The University of Tokyo, Tokyo, 113-8655 Japan

**Keywords:** Myopic choroidal neovascularization, Polarization-sensitive optical coherence tomography, Polarimetric entropy, Melanin, Encapsulation, Fuchs’ spot

## Abstract

**Purpose:**

We aimed to investigate the association between post-treatment changes in polarimetric entropy (PE), which is a noninvasive surrogate marker of structural heterogeneity associated with pigment epithelial remodeling and measured by polarization-sensitive optical coherence tomography (PS-OCT), and the need for multiple anti-vascular endothelial growth factor (VEGF)-injections in myopic choroidal neovascularization (mCNV).

**Methods:**

We retrospectively analyzed 11 eyes that received intra-vitreal anti-VEGF injections for treatment-naïve mCNV. Based on treatment response during the first 3 months, eyes were categorized into the single-injection (*n* = 9) and multiple-injection (*n* = 2) groups. PS-OCT en face PE maps (6 × 6 mm) were obtained at baseline and 3 months after treatment, with the analysis area manually centered over the CNV using a 3-mm Early Treatment Diabetic Retinopathy Study (ETDRS) grid. PE values were extracted from the central 1-mm circle (CNV core) and surrounding 1–3-mm annulus (periphery), and the differences before and after treatment were calculated. Intergroup comparisons were conducted using Wilcoxon rank-sum test.

**Results:**

PE within the CNV core increased after treatment (*p* = 0.0037). The multiple-injection group showed a greater PE increase than the single-injection group (*p* = 0.0347), although this finding should be interpreted cautiously given the small and imbalanced sample size. Exploratory analysis suggested a positive association between the magnitude of PE change in the CNV core and injection number during the 3-month period.

**Conclusions:**

High post-treatment entropy may reflect structural heterogeneity associated with pigment epithelial remodeling during the early healing phase. These findings suggest that PS-OCT entropy imaging may serve as a retrospective indicator of tissue response rather than a predictive biomarker in the present dataset. Given the very small and imbalanced sample size, the results should be interpreted as exploratory and hypothesis-generating.

**Supplementary Information:**

The online version contains supplementary material available at https://doi.org/10.1186/s40942-026-00902-6.

## Key Message

**What is already known on this topic**

・Conventional OCT is limited in evaluating the maturity or extent of reparative tissue (e.g., Fuchs spot-like encapsulation) in myopic choroidal neovascularization (mCNV).

**What this study adds**

・This is the first study to quantitatively assess structural heterogeneity associated with centripetal remodeling of mCNV lesions using polarization-sensitive OCT (PS-OCT)–derived entropy imaging.

・Eyes requiring multiple anti-VEGF injections exhibited greater post-treatment entropy increases within the lesion core, suggestive of more pronounced and heterogeneous pigment epithelial remodeling.

・PS-OCT enables noninvasive monitoring of tissue remodeling dynamics in mCNV and offers novel insights into the biological underpinnings of lesion regression and treatment responsiveness.

**Supplementary Information:**

The online version contains supplementary material available at https://doi.org/10.1186/s40942-026-00902-6.

## Introduction

Pathologic myopia is the most common cause of irreversible visual impairment, especially in East Asian populations. Myopic choroidal neovascularization (mCNV) is one of its most vision-threatening complications, frequently affecting relatively young patients and causing rapid central vision loss [1].Intravitreal anti-vascular endothelial growth factor (anti-VEGF) therapy has improved the visual prognosis of mCNV [2]. However, recurrence remains a major challenge, occurring in approximately 30%–50% of eyes within the first year after treatment initiation [3, 4]. Recurrence often requires repeated injections, which may accelerate chorioretinal atrophy and fibrotic scar formation and ultimately contribute to long-term visual decline.

Several studies have sought to identify recurrence risk factors, including CNV size, retinal pigment epithelium (RPE) alterations, number of anti-VEGF injections, and presence of perforating scleral vessels [5–7]. These factors offer structural and clinical information but do not capture the biological characteristics or maturation status of healing tissue Histologic studies have shown that during the healing process, mCNV can become encapsulated by melanin-containing tissue derived from RPE cells (i.e., Fuchs’ spot) [8]. Similarly, indocyanine green angiographic studies on idiopathic CNV have shown that a hypofluorescent dark rim surrounding the neovascular membrane, which is thought to represent proliferated RPE, correlated with lesion regression and decreased exudation, providing in vivo evidence of the role of RPE-derived encapsulation in stabilizing CNV [9]. However, conventional optical coherence tomography (OCT) is limited in its ability to visualize and assess the structural quality and uniformity of the reparative tissue that encapsulates the mCNV lesion (e.g., Fuchs spot-like hyperpigmented scarring). Polarization-sensitive OCT (PS-OCT) is a new imaging modality that allows visualization of tissue-specific polarization properties, such as birefringence and depolarization, and detection of fibrous and melanin-rich structures that are invisible on standard OCT [10]. Among its analytical methods, entropy mapping quantifies the spatial randomness of polarization states and serves as an indicator of tissue heterogeneity. Ahn et al. reported that subretinal fibrosis after anti-VEGF therapy was associated with recurrence and poor visual outcomes; however, the biological maturity and structural organization of reparative tissue remain difficult to evaluate using conventional OCT [11]. PS-OCT has the unique capability to detect melanin-associated depolarization signals and quantify their spatial heterogeneity using entropy analysis [10]. Previous PS-OCT studies have shown that polarimetric entropy can quantify RPE melanin distribution in highly myopic eyes and is associated with retinal sensitivity, supporting its relevance in the myopic fundus [12]. In addition, recent longitudinal PS-OCT studies demonstrated that anti-VEGF therapy can induce short-term changes in RPE melanin distribution in neovascular AMD [13] and that PS-OCT can visualize remodeling of RPE-derived repair tissue over time [14]. These findings provide the rationale for applying PS-OCT-derived entropy analysis to evaluate post-treatment structural heterogeneity associated with pigment epithelial remodeling in mCNV [14]. Because RPE-derived tissue remodeling and melanin redistribution are key components of the healing process following anti-VEGF treatment in mCNV [8, 9], we hypothesized that PS-OCT–based entropy analysis may capture tissue-level remodeling after treatment and provide additional biological context for interpreting the early treatment course.

In this study, we used PS-OCT entropy mapping to quantify structural heterogeneity associated with RPE-related remodeling in mCNV lesions before and after anti-VEGF treatment. Next, we examined the difference in PE changes between single and multiple anti-VEGF injections within the initial 3-month period.

## Methods

### Study design and participants

This retrospective study was approved by the institutional review board of the University of Tokyo (Ethics Committee ID number: 11822), which waived the requirement for written informed consent, and adhered to the tenets of the Declaration of Helsinki. All data were fully anonymized before being accessed, and participants who did not grant authorization to use their medical records for research were excluded from analyses.

Clinical trial number: not applicable.

We retrospectively reviewed the medical history of patients with mCNV who received once or twice intra-vitreal anti-VEGF injections from April 2023 to November 2024 at the outpatient clinic of Tokyo University Hospital. All patients were examined on their visit after 3 months. The inclusion criteria were (1) high myopia, defined as axial length ≥ 26.5 mm; (2) active mCNV confirmed by multimodal imaging; and (3) availability of high-quality PS-OCT images at baseline and 3 months after initial treatment. Active mCNV was defined as the presence of a neovascular lesion detected on spectral-domain OCT with Enhanced Depth Imaging (Heidelberg Engineering GmbH, Dossenheim, Germany) and OCT angiography corresponding to exudative findings (subretinal and/or intraretinal fluid) or hemorrhagic changes on structural OCT. Spectral-domain OCT and OCT angiography were performed in all eyes, fluorescein angiography was performed unless contraindicated, and indocyanine green angiography was obtained when clinically indicated.

### Treatment protocol and groups

All patients received one intra-vitreal injection with either ranibizumab (0.5 mg) or aflibercept (2.0 mg), followed by additional injections as needed for clinical and imaging findings, such as subretinal hemorrhage, serous retinal detachment, and subretinal hyperreflective materials. Based on the number of anti-VEGF injections administered during the first 3 months, patients were categorized into the single-injection and multiple-injection (≥ 2) groups. During the available 12-month clinical follow-up, no additional injections were recorded after the initial 3-month treatment period in the included eyes.

### PS-OCT imaging and entropy analysis

PS-OCT imaging was performed at baseline and 3 months after injection using a prototype swept-source system, which had a center wavelength of 1050 nm and was equipped with continuous source polarization modulation^10^. For image analysis, a modified ETDRS grid was used to define two circular regions on the center of the CNV lesion: a 1-mm central circle and a 1–3-mm inner ring. CNV area was defined as the region within a 1-mm radius directly overlying the CNV lesion, and the peripheral region was defined as the surrounding area 1–3 mm from the CNV. Examiners were masked to injection number and clinical outcomes during entropy measurement. The 1-mm central circle was selected as a standardized lesion-centered ROI rather than a manually traced CNV boundary. This approach was chosen to improve reproducibility and comparability across eyes by using a fixed ETDRS-based region centered on the CNV lesion. In all included eyes, the clinically identifiable CNV lesion was contained within the central 1-mm circle. The surrounding 1–3 mm annulus was used as an internal reference region to account for inter-eye and inter-scan variability in baseline entropy signals.

After automatic segmentation, experienced examiners (MO and KA) manually refined the segmentation lines by referring to intensity images. A 3-pixel-thick RPE slab centered on the RPE line was generated, and en face entropy maps were created by averaging entropy values across A-lines within this slab.

Given the axial resolution of the PS-OCT system (4.4 μm per pixel), a 3-pixel slab corresponds to approximately 13 μm, closely approximating the known anatomical thickness of the RPE. This thickness was selected to capture the full RPE layer while minimizing contamination from adjacent structures such as Bruch’s membrane and the photoreceptor outer segments. In addition, averaging entropy values across this thin slab reduces the influence of speckle noise and mitigates minor segmentation variability, thereby improving measurement stability.

**Fig. 1:**
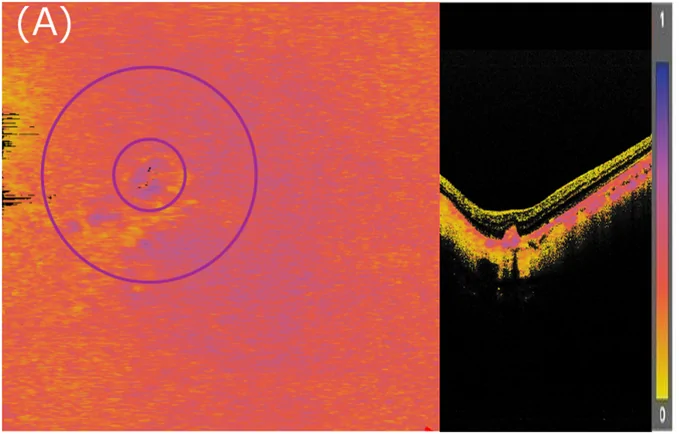
(**a**) En face entropy image at the RPE level, with automated extraction of 1- and 3-mm circular ROIs used to quantify and normalize polarimetric entropy within the lesion and its surrounding tissue. (**b**) B scan PS-OCT image displays the corresponding entropy distribution

Sensitivity analyses were performed using ± 1-pixel variations (2- and 4-pixel slabs). These analyses yielded comparable entropy values, and the direction and statistical significance of intergroup differences remained unchanged, confirming the robustness of the slab selection. Entropy values, derived from melanin-induced depolarization signals, range from 0 (uniform polarization state) to 1 (maximally random polarization distribution). Entropy was quantified within two concentric regions of interest (ROIs): a 1-mm central circle (CNV core) and a surrounding 1–3 mm annulus (peripheral ring) (Fig. 1). Entropy within the CNV core was expressed relative to the surrounding 1–3 mm annulus to reduce inter-scan variability. No additional pixel-level thresholding was applied; averaging across the defined slab was used to mitigate local noise influence. To evaluate treatment-induced changes, we defined the entropy ratio as the PE value in the CNV core divided by the PE value in the peripheral ring. The change in entropy ratio (Δ entropy ratio), which reflected the increase in CNV core PE relative to the peripheral ring, was calculated as follows:

Δ entropy ratio = entropy ratio at 3 months − entropy ratio at baseline.

Inter-observer reproducibility of entropy measurements was assessed in all eyes using intraclass correlation coefficient (ICC).

### Outcome measures

The primary outcome was the association between Δ entropy ratio and number of injections received at 3 months (single vs. multiple). Secondary analyses included changes in the CNV core entropy and visual acuity from baseline to 3 months after treatment and correlation between entropy parameters and injection number. Best corrected visual acuity (BCVA) was measured in logarithm of the minimum angle of resolution (logMAR) units.

### Statistical analysis

Statistical analyses were conducted using JMP Pro software version 17 (SAS Institute, Cary, NC, USA). Entropy change was compared between groups using Wilcoxon rank-sum test, and correlations between entropy change and number of injections were analyzed using Spearman’s rank correlation coefficient. A two-sided p value of < 0.05 was considered statistically significant.

## Results

A total of 11 eyes from 11 patients (mean age 66.0 ± 18.2 years; 3 men and 8 women) with mCNV were included. The mean axial length was 28.9 ± 1.91 mm. Table 1 shows a summary of demographic and clinical characteristics of the study eyes. Seven eyes were phakic, and four were pseudophakic. The mean baseline BCVA was 0.45 ± 0.19 logMAR, which improved to 0.23 ± 0.21 logMAR at 3 months post-treatment. The initially administered intra-vitreal anti-VEGF therapy was ranibizumab in 8 eyes (72.7%) and aflibercept in 3 eyes (27.3%).

**Table 1:** Baseline characteristics of the study eyes (*n* = 11)

Parameters	All eyes (*n* = 11)
Age (years)	66.0 ± 18.2
Male/Female (%)	3/8 (27.3% / 72.7%)
Phakia/IOL	7/4
Axial length (mm)	28.9 ± 1.91
Pre BCVA (logMAR)	0.45 ± 0.19 (range 0.15–0.70)
Post BCVA (logMAR)	0.23 ± 0.21 (range − 0.01–0.52)
Anti-VEGF (n, %)	
Ranibizumab	8 (72.7%)
Aflibercept 2 mg	3 (27.3%)

**Fig. 2:**
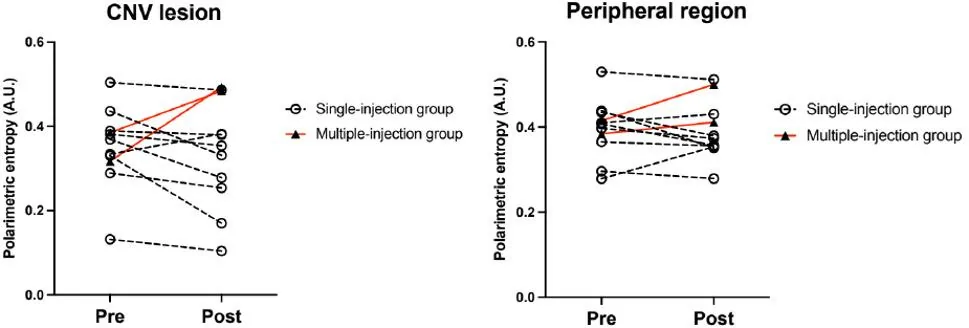
Line plots of the individual entropy values at baseline and 3 months post-treatment. In the multiple-injection group, there is increased entropy in the CNV core (left) during the initial treatment phase in both eyes (red lines). In contrast, entropy changes in the surrounding perifoveal area (right) are less consistent and not clearly associated with injection frequency

Table 2 summarizes the BCVA outcomes over time. The single‑injection group (*n* = 9) demonstrated a mean improvement in BCVA of ‑0.17 ± 0.16 logMAR, from 0.39 ± 0.15 logMAR at baseline to 0.22 ± 0.23 logMAR at 3 months. The multiple‑injection group (*n* = 2) showed a greater mean BCVA improvement of ‑0.39 ± 0.12 logMAR, from 0.70 ± 0.00 logMAR at baseline to 0.31 ± 0.12 logMAR at 3 months. Both eyes in the multiple-injection group received two anti-VEGF injections during the initial 3-month treatment period; no eye required three or more injections during this interval. A clinically meaningful visual gain (≥ 0.2 logMAR) was observed in 4 of 9 eyes (44.4%) in the single‑injection group and in all eyes (100%) in the multiple‑injection group. On comparison of the polarimetric entropy (PE) values before and at 3 months after treatment (Fig. 2), there was a consistent post-treatment increase in entropy in the CNV core and peripheral ring in the multiple-injection group. The ICC for CNV core entropy was 0.85 (95% CI: 0.79–0.92), indicating good reproducibility. Entropy measurements derived from 2-, 3-, and 4-pixel slabs showed comparable values and preserved intergroup differences, indicating stability of the quantification.

**Table 2:** Visual acuity outcomes in the single- and multiple-injection groups

	At baseline	At 3 months	At 1 year
Single-injection group (*n* = 9)	0.39 ± 0.15	0.22 ± 0.23	0.16 ± 0.20
Multiple-injection group (*n* = 2)	0.70 ± 0	0.31 ± 0.12	0.08 ± 0.11

Values are presented as mean ± SD

**Fig. 3:**
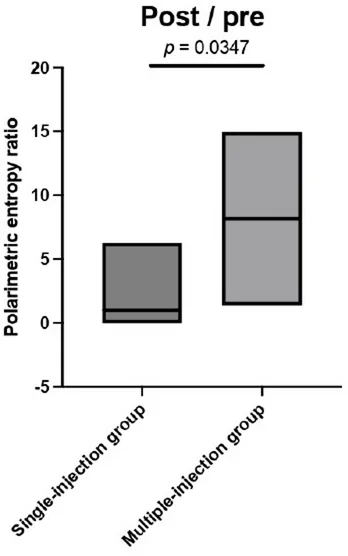
Eyes in the Multiple-injection group showed a greater increase in entropy ratio compared to those in the Single-injection group (*p* = 0.0347, Wilcoxon rank-sum test)

**Fig. 4:**
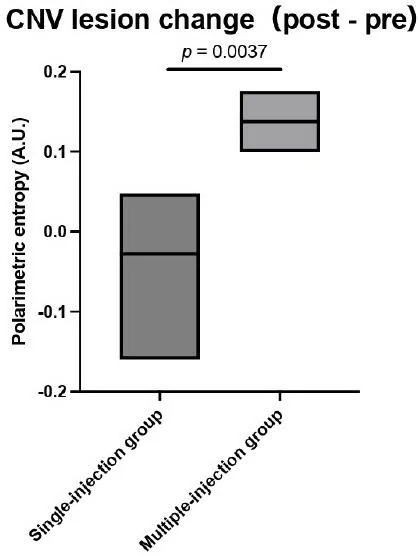
Box plot of the changes in polarimetric entropy within the central CNV lesion between baseline and 3 months post-treatment. Entropy change showed a greater increase in the multiple-injection group than in the single-injection group (p = 0.0037, Wilcoxon rank-sum test)

At 3 months after initial anti-VEGF treatment, Δ entropy ratio was higher in the multiple-injection group than in the single-injection group (*p* = 0.0347), corresponding to a large effect size (*r* = 0.60) (Fig. 3). Further analysis focusing on the CNV core showed a greater increase in absolute PE values (post–pre) in the multiple-injection group than in the single-injection group, with a large effect size (*r* = 0.60). Although the difference reached statistical significance (*p* = 0.0037), it should be interpreted cautiously given the exploratory nature and small sample size (Fig. 4). As an exploratory descriptive analysis, a positive association was observed between the magnitude of entropy change within the CNV core and the number of anti-VEGF injections administered during the early treatment phase (Spearman’s *r* = 0.79, 95% CI: 0.37–0.94; *p* = 0.0037). However, because injection frequency showed very limited variability, with most eyes receiving only a single injection, this correlation should be interpreted with caution (Supplementary Fig. S1).

**Fig. 5:**
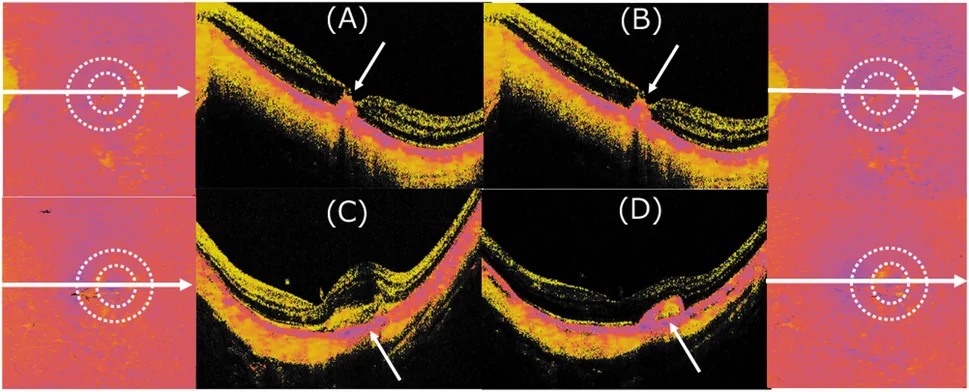
Representative en face PS-OCT entropy maps. (**A**) Baseline image from a representative eye in the single-injection group. (**B**) Three-month follow-up image from the same eye. (**C**) Baseline image from a representative eye in the multiple-injection group. (**D**) Three-month follow-up image from the same eye. Warmer colors indicate higher entropy values. Eyes in the single-injection group exhibit relatively mild increases in entropy while maintaining relatively homogeneous polarization patterns. In contrast, eyes in the multiple-injection group show more pronounced entropy elevation and patchy irregular polarization textures within the lesion core, consistent with greater structural heterogeneity

Figure 5 shows representative PS-OCT images at baseline and 3 months after treatment. Eyes in the single-injection group showed only mild changes in entropy values, with relatively uniform polarization patterns. On the other hand, eyes in the multiple-injection group exhibited markedly increased entropy and irregular heterogeneous polarization patterns at 3 months, indicating more extensive remodeling within the CNV core. These findings likely reflected a reactive remodeling process associated with greater tissue heterogeneity, which was observed in eyes requiring multiple injections during the early phase. Notably, such tissue-level changes were not detectable by conventional OCT, highlighting the unique utility of PS-OCT in visualizing post-treatment structural heterogeneity. During clinical follow-up through 12 months, no additional anti-VEGF injections were recorded after the initial 3-month treatment period in the included eyes. However, 12-month PS-OCT imaging was not available for all eyes; therefore, longitudinal entropy changes beyond 3 months were not analyzed.

## Discussion

This study examined whether post-treatment changes in PE, which was measured by PS-OCT as a potential surrogate for structural heterogeneity associated with pigment epithelial remodeling, were associated with the early need for multiple injections in mCNV. In our cohort, eyes that subsequently required multiple injections within 3 months showed greater post-treatment increase in PE, particularly within the CNV core, suggesting more pronounced pigment epithelial remodeling in eyes requiring repeated treatment. These results demonstrated that increased PE reflected greater structural heterogeneity associated with pigment epithelial remodeling after anti-VEGF therapy. Given the small and imbalanced sample and the relatively wide confidence interval, these findings should be interpreted with caution and regarded as hypothesis-generating rather than confirmatory.

PE quantifies the spatial randomness of polarization states in PS-OCT and is closely linked with the heterogeneity of melanin-associated depolarization. Melanin granules within the RPE and RPE-derived fibrous tissue cause depolarization of incident light, making PE a sensitive metric of local variations in melanin content and distribution [10]. Elevated entropy likely reflects spatially heterogeneous melanin distribution and dynamic remodeling of RPE-derived tissue during the healing phase, rather than definitively indicating either mature or failed encapsulation. Histological studies have shown that successful pigment epithelial encapsulation of mCNV lesions involves orderly migration and proliferation of RPE-derived cells with progressive accumulation of melanin granules and collagenous matrix [8–11, 15]. The resulting Fuchs’ spots, which are typically found in long-standing nonexudative lesions, comprise stable and melanin-rich fibrous tissue that prevents CNV reactivation [11, 15]. Conversely, irregular hypertrophic fibrosis with incomplete encapsulation may fail to constrain neovascular proliferation and lead to recurrent exudation [11]. Other histopathological studies on highly myopic eyes have revealed that choroidal neovascular membranes are often surrounded by heterogeneous fibrous tissue with variable melanin deposition, along with degenerative changes, such as atrophic or fragmented RPE and discontinuous Bruch’s membrane [16]. The findings by Grossniklaus and Green also emphasize the structural fragility in pathologic myopia, which may predispose the macula to secondary complications such as CNV [16]. In our case, the irregular PE pattern might reflect such histologic heterogeneity—ranging from heterogeneous fibrous tissue to melanin-containing remnants—surrounding the neovascular complex. These observations suggested that elevated PS-OCT entropy reflects ongoing remodeling or reparative changes at the RPE level, particularly in eyes with more active disease biology, and may provide information beyond what is captured by conventional structural imaging.

Although PS-OCT imaging was not performed at the time when additional treatment was required, our data showed that greater PE at 3 months post-treatment (i.e., healing phase) was associated with the need for multiple injections during that period. This greater PE elevation at 3 months may have reflected a consequence of tissue-level remodeling in eyes that required repeated therapy, rather than a factor that directly triggered the need for further treatment. In this context, the elevated PE at 3 months in eyes that required multiple injections likely reflected more extensive and heterogeneous pigment epithelial remodeling that developed as part of the biological course and increased early treatment demand, rather than a subclinical activity per se [9, 15, 16]. Moreover, PS-OCT enables depth-resolved, tissue-specific analysis of lesion remodeling [10]. When exudation resolves, intensity-based OCT struggles to differentiate fibrous tissue from surrounding structures, whereas PE reflects melanin-associated signals independent of reflectivity or fluid status. This allows more precise delineation of entropy-defined tissue heterogeneity at the cellular and subcellular levels. Thus, PE may provide additional information on tissue-level structural heterogeneity beyond conventional OCT. These findings may provide additional biological context for interpreting post-treatment structural changes in mCNV, which largely rely on signs of exudation or subjective visual complaints.

In this study, PE elevation at 3 months after treatment—specifically, an increase in polarimetric entropy within the central CNV lesion—was predominantly observed in eyes that required multiple injections during the early treatment phase. Further studies are warranted to determine how this entropy-based parameter relates to lesion status in prospective longitudinal cohorts. In addition, all eyes were structurally dry during evaluation at 3 months, reflecting the fact that PE was evaluated during a quiescent healing phase rather than during exudative reactivation or prior to additional treatments. However, we found that eyes with greater increase in PE within the CNV core within the initial 3 months required multiple injections during that period. This implied that PE elevation may not have preceded reactivation but rather reflected tissue remodeling processes due to more active disease biology [9, 15, 16]. In this sense, PE may serve as a retrospective indicator of tissue response dynamics in eyes that require more intensive treatment. We observed a consistent post-treatment increase in the central-to-peripheral PE ratio. Although speculative, this spatial pattern may suggest regionally heterogeneous remodeling around the CNV lesion and should be further examined in larger longitudinal studies [14].

The previously identified clinical and imaging-based risk factors for treatment burden in mCNV include greater lesion size, juxtafoveal location, irregular RPE morphology, and higher injection requirements during the initial treatment phase [5–7]. Notably, Jain et al. reported that eyes requiring ≥ 3 injections during the first episode had a 4-fold greater likelihood of requiring retreatment [7]. Our findings complement these observations by introducing PE as a quantitative microstructural metric that captures tissue-level remodeling dynamics beyond conventional structural imaging. In the future, integrating PE with conventional clinical and imaging factors may contribute to a broader framework for understanding tissue-level treatment response in mCNV. PS-OCT–derived PE is a novel biologically grounded imaging metric that reflects tissue-level remodeling dynamics in mCNV [10]. It potentially captures qualitative differences in melanin‑rich tissue organization, which are not visible on conventional OCT, and can provide insights into the wound healing status of the RPE-choroid interface. An interesting observation in our cohort was that none of the eyes required additional injections between 3 and 12 months. However, this observation should be interpreted cautiously because 12-month PS-OCT imaging was not available for all eyes.

There were several limitations in this study. First, the sample size was small and drawn from a single tertiary center. Given the very small and imbalanced sample, the study was underpowered to support definitive inferential conclusions. While the observed group difference showed a large effect size (*r* = 0.60), interpretation should be cautious given the limited sample size. Although the overall trends were internally consistent, external validation in larger prospective multicenter cohorts is necessary. In addition, patients were not selected according to disease severity or treatment response. Rather, this retrospective study included all consecutive eyes that met the predefined eligibility criteria, including the availability of high-quality PS-OCT images at baseline and 3 months. Nevertheless, selection bias inherent to these retrospective inclusion criteria cannot be excluded. This cohort may not fully represent the entire spectrum of treatment-naïve mCNV encountered in routine clinical practice. Therefore, the absence of additional injections between 3 and 12 months should be interpreted only within the context of the analyzed cohort and should not be generalized to all patients with mCNV. In addition, the Spearman correlation analysis was limited by the restricted variability in injection frequency, as most eyes received only a single injection. Therefore, the observed correlation may have been strongly influenced by the small number of eyes requiring repeated injections and should be regarded as exploratory rather than confirmatory. Second, entropy analysis required manual refinement of segmentation and ROI positioning. Inter-observer reproducibility was assessed in a subset of eyes and demonstrated good agreement (ICC = 0.85, 95% CI: 0.79–0.92). This supports the robustness of entropy quantification despite manual refinement. The relatively high reproducibility may be attributable to the standardized ROI definition using the ETDRS grid, in which the 1-mm central circle encompassed the entire mCNV lesion in all cases. Furthermore, entropy values were averaged within predefined regions, reducing sensitivity to minor variations in segmentation. Although the ETDRS-based ROI approach improved reproducibility, it did not account for individual variations in CNV lesion size or morphology. Therefore, entropy values may have been influenced by the relative proportion of CNV tissue and surrounding tissue within the fixed central ROI. Future studies using lesion-specific segmentation or size-adjusted ROI analyses are needed to validate the present findings. Third, the biological basis of entropy increase remains to be clarified. Although all included eyes had clinical follow-up data through 12 months, 12-month PS-OCT imaging was not available in all cases. Therefore, we could not evaluate whether PE values remained stable, increased, or decreased after the 3-month healing phase. This limits interpretation of the temporal evolution of entropy changes and should be addressed in future prospective studies with standardized longitudinal PS-OCT imaging. Finally, although exploratory associations were observed, the biological basis of entropy increase, whether driven by fibrosis dynamics, inflammatory activity, RPE integrity, or vascular remodeling, remains to be elucidated. Future studies integrating PS-OCT with molecular imaging or histopathology would help clarify the pathophysiological mechanisms. Increased PE likely reflects microstructural heterogeneity in melanin-associated depolarization signals, consistent with dynamic pigment epithelial remodeling during early healing. In the present dataset, PE should not be interpreted as a direct indicator of encapsulation maturity or quality. In this context, PE elevation should be interpreted as reflecting dynamic pigment epithelial remodeling during the early healing phase rather than serving as a direct surrogate of encapsulation quality. We hypothesize that entropy may increase during early dynamic remodeling due to heterogeneous melanin redistribution and gradually stabilize as tissue remodeling becomes more organized over time. Further longitudinal studies are required to confirm this temporal evolution.

## Conclusions

Our study showed that increased entropy, particularly within the CNV core, was associated with the need for multiple injections during the initial treatment phase. These findings suggest that PE may reflect dynamic and heterogeneous pigment epithelial remodeling during early healing rather than a direct indicator of encapsulation maturity. In this small exploratory cohort, PS-OCT–derived entropy should be interpreted as a retrospective indicator of tissue response, and prospective studies are needed to clarify its potential clinical implications.

## Supplementary Information

Below is the link to the electronic supplementary material.

Supplementary Material 1: Figure S1: Scatter plot showing the relationship between the magnitude of normalized CNV core entropy change and the number of anti-VEGF injections administered during the initial 3-month treatment period. Each dot represents one eye. The analysis is exploratory because only two eyes required two injections during the study period

## Data Availability

The datasets generated and/or analyzed during the current study are not publicly available due to institutional and ethical restrictions on sharing clinical imaging data, but are available from the corresponding author upon reasonable request.

## Declarations

### Data transparency

No public dataset or repository was used. All data supporting the findings of this study were obtained at the University of Tokyo Hospital and are available from the corresponding author upon reasonable request.
